# Historicising the prohibition of vulval cutting in the UK: how race and medicine shaped the criminalization of female genital mutilation

**DOI:** 10.1093/medlaw/fwag034

**Published:** 2026-09-29

**Authors:** Mireia Garcés de Marcilla

**Affiliations:** School of Law and Social Justice, University of Liverpool, Liverpool, L69 7ZR, United Kingdom

## Abstract

This article is a historical analysis of the law governing vulval cutting in the UK, with a particular focus on the shifting role and perception of the medical profession in this context. It shows how criminalization is not neutral or inevitable, but a strategy that developed through historically contingent negotiations between law and medicine. It charts how racially laden ideas of health, expertise and medical legitimacy informed successive legislative reforms. In doing so, it uncovers the genesis of how racialized understandings of medical authority and mental health were first used to draw fuzzy lines between lawful and unlawful operations, and argues that subsequent concerns of vulval cutting as a widespread problem within migrant communities displaced earlier debates about the contours of the medical exception, cementing its imprecise boundaries. In turn, this article suggests that, during these 40 years of legislative development, the role of doctors has changed considerably, from shaping the limits of the criminal prohibition to being responsibilized for enforcing it.

The summer of 2025 marked the 40-year anniversary of Parliament passing the Prohibition of Female Circumcision Act 1985 (henceforth, the 1985 Act). This Act made it an explicit criminal offence ‘to excise, infibulate or otherwise mutilate the whole or any part of the labia majora or labia minora or clitoris of another person’,^1^ saving for ‘necessary surgical operations’.^2^ The Female Genital Mutilation Act 2003 (henceforth, the 2003 Act) replaced the 1985 Act, and the Serious Crime Act 2015 strengthened and expanded the scope of the provisions in the 2003 Act. This article maps the history—or histories—of these three key legislative moments in the law governing vulval cutting in the UK.^3^ It provides a ‘biography’ of the prohibition of ‘female circumcision’ or ‘female genital mutilation’ (FGM), tracing the origin and evolution of the regulation of this practice with a particular focus on the shifting role and perception of the medical profession in this context.

Rather than offering an account on how the prohibition emerged in a particular context, this article uses ‘legal biography’ as a method to present a ‘historical, contextual study’ of the law on vulval cutting as a ‘continuing and evolving whole, which is always in the process of accumulating meaning’.^4^ It assesses ‘moments of challenge, rupture, and revision’ of this legal framework, whilst reflecting about ‘the shifting broader social and institutional contexts within which [it] is read, understood and applied’.^5^ In charting the themes that have emerged, persisted, or receded in the last 40 years, this article sheds new light on the relationship between criminalization, race, and medical authority in the regulation of vulval cutting. Far from being a neutral or inevitable response to harm, this article argues that criminalization developed through historically contingent negotiations between law and medicine. It traces how racially laden ideas of health, expertise, and medical legitimacy informed successive legislative reforms. In doing so, it uncovers how racialized understandings of mental health are used to draw fuzzy lines between lawful and unlawful cuts, while tracking the ways in which medical professionals participate in delimiting the scope of the criminal law, whilst also having become important actors within the criminal justice system.

Several scholars have examined the history of the prohibition of vulval cutting in some former British colonies,^6^ but a contemporary history of the legislation of the practice in the UK has received little attention.^7^ Some accounts present a ‘snapshot’ of particular legislative moments,^8^ or examine specific measures in relation to vulval cutting.^9^ Lately, academic writing has also been devoted to the slow judicial engagement with vulval cutting, including care proceedings,^10^ protection orders,^11^ and the recent upwards trajectory in the number of prosecutions.^12^ Feminist engagements with this topic are extensive, and have tackled questions such as cultural relativism and Western imperialism,^13^ the oppressive nature of the practice,^14^ and the role of law and human rights in deeming this intervention illegitimate.^15^ In the British context, as this article examines, this discussion has translated into a lengthy debate about the division between ‘medical’ or ‘cultural’ vulval cutting.^16^ This article chronicles the genesis of this distinction, arguing that it must be found in the desire of respecting the autonomy of the medical profession in performing vulval operations.

Although this article focuses on the role of race and medical authority in shaping legislative developments, other factors have also influenced reform. Indeed, the UK legal framework must be located within broader international trends whereby vulval cutting for traditional or religious reasons has been deemed a human rights violation which requires legal but also ‘multisectoral, sustained and community-led’ action, via education, healthcare and nongovernmental organization.^17^ The Women and Equalities Committee highlighted in 2025 that law is not the only strategy to tackle vulval cutting, which should also include access to healthcare and counselling services,^18^ and education and awareness rising campaigns.^19^ Therefore, medical professionals are not the only relevant stakeholders in this context, as other actors, most notably grassroots organizations,^20^ have played an important role in shaping legislation.^21^ Yet, this paper devotes most of its attention to the medical profession, since not only has it been very influential since the first prohibition was enacted, but also because its perception and role has changed considerably since 1985. This article suggests that concerns about medical freedom and exclusionary notions of race and medical expertise were key for how the first prohibition was enacted in 1985, assessing how these considerations have evolved until the last round of reforms in this area.

Section I shows how the media framing of vulval cutting as both clandestine and medically sanctioned foregrounded an early tension that would come to characterize the 1985 Act: the absence of conceptual clarity as to what, precisely, was being prohibited. As this section demonstrates, the legislative response emerged in a context of definitional uncertainty, in which anxieties about professional medical conduct, cultural practices, and bodily harm were collapsed into a criminal prohibition whose boundaries were left imprecise. Section II argues that the 2003 Act maintained the fuzziness whilst expanding the reach of the criminal prohibition extraterritorially, which was prompted by a shift in the perception of vulval cutting from a ‘rare’ occurrence within the medical sphere to a widespread problem amongst rising immigrant communities. Section III spells out the ways in which 2015 reforms widened criminalization, revealing major changes with regard to medical professionals, whose role as quasi-legal actors evolved from shaping the contours of the criminal prohibition to being responsibilized for enforcing it. The last round of reforms also exposes the perceived anxiety about the penal machinery set in place for FGM—which now features several offences, civil-criminal hybrid remedies, and reporting duties for medical professionals—not having successfully translated into a rise in convictions. Since 1985, there have been only been five prosecutions for FGM, all in the last decade: the first prosecution was brought in 2015, which was unsuccessful, and another failed prosecution followed in 2018.^22^ The first conviction dates from 2019,^23^ followed by another one in 2023,^24^ and the last one thus far in 2024.^25^ Only one of these cases has been reported, since, as expanded upon below, its sentencing was appealed to the Court of Appeal. As this article covers the span of time starting with the first statutory prohibition until the last package of legislative reforms, prosecutions do not play a central role in its analysis given that, except for the first one, the rest of FGM criminal cases are post-2015.

The concepts of ‘race’ and ‘racialization’ have multiple and contested meanings,^26^ and this article sees ‘racialization’ as a process of production of racial bodies and identities.^27^ Thus, it analyses how vulval cutting has been constructed as an issue of ‘race’ and, more precisely, as a harmful practice that must be criminalized and expelled from the realm of medicine. Importantly, this article does not suggest that some forms of vulval cutting are not harmful (the WHO has gathered a long list of negative side effects, including bleeding, pain, risk of infections, psychological trauma, and even death^28^), or that nothing should be done to protect those vulnerable to harm. Instead, this article unravels how criminalization became ‘vital’ in the UK for tackling this intervention.^29^ The use of nomenclature when talking about vulval ‘cutting’, ‘circumcision’, or ‘mutilation’ is also contested and has a history: ‘female circumcision’ was the main term in usage until the early 1990s, when, as Section II explains, ‘FGM’ started to gain popularity. This article uses the terms ‘circumcision’ and ‘mutilation’ chronologically, deploying the former when referring to law pre-2003 (when the statute adopted the FGM terminology) and the latter when discussing post 2003 developments.

## I. The Prohibition of Female Circumcision Act 1985

### A. Media exposure and legal fuzziness

In October 1982, *The Observer* published an investigation revealing that ‘female “circumcisions” [were] being carried out in private London clinics up to £1000 a time’.^30^ A Harley Street-based doctor, Sunit Ghatak, admitted to the newspaper that he had performed these operations on two patients.^31^ Both were of Nigerian origin; the first patient was persuaded by her husband that she could not conceive otherwise. The second one, who had already given birth to a baby via a Caesarean section, believed the intervention was necessary to be able to vaginally deliver any potential future children.^32^ Gathak did not believe circumcision would make a difference for fertility or how these patients had children, but agreed to it because it could have a ‘psychological’ benefit^33^: ‘I do not encourage the operation because there is no medical reason or advantage, but if someone comes in because of suffering depression and all that—then we do it’.^34^

A few days after this news broke, the British Medical Association (BMA) and the General Medical Council (GMC) declared that they were ‘powerless’ to stop Harley Street surgeons from performing these interventions, explaining that ‘not only is the practice legal in Britain, but the GMC would have to prove that the operation was carried out on non-medical grounds before it could take disciplinary action’.^35^ The BMA deferred to the GMC to ‘take the lead in giving guidelines to doctors’, and the GMC responded that they could not act unless a patient, doctor, or organization would bring the issue in front of the council as a matter of ‘professional ethics’ via a ‘letter of concern’.^36^ The Government deflected responsibility as well, considering that it could not ‘interfere with clinical judgement’ and that ‘if a doctor says there is a medical reason for the operation, we have to accept it’.^37^ A month later, Kenneth Clarke, who was then the Minister of State for Health, maintained this position in his written answer in Parliament: while condemning the ‘abhorrence’ of the practice, he insisted that ‘unethical practices by doctors are a matter for the GMC to consider’, and that the Government would not take any action before hearing from the GMC first.^38^

Lord Kennet, the Social Democratic Party’s Chief Whip in the House of Lords, had already raised female circumcision as an issue in Parliament a few months prior, asking the Government about medical and nursing staff training working abroad,^39^ and support programmes to ‘educate’ people against the practice.^40^ When the story about Ghatak broke, he harnessed media attention, telling *The Observer* that if ‘there [was] no law against [female circumcision], there was certainly a case for introducing one’.^41^ He was not the only one who had doubts about the legal status of the practice. The GMC declarations quoted above suggested that the practice was legal, and Norman Fowler, the Secretary of State for Social Services, could not provide a clear answer as to whether it was illegal or not, explaining that, since ‘there is no specific legislation which prohibits it … the legal position is less clear’.^42^ There was reticence on behalf of the Government to introduce legislation to clear the fuzziness, given the challenge of outlawing female circumcision without encroaching on legitimate surgical interventions, such as removal of cancerous tissue.^43^ Eventually, Lord Kennet introduced the Prohibition of Female Circumcision Bill as a Private Member’s Bill,^44^ using Ghatak’s story in his speech,^45^ which also appeared in a BBC 40 minutes programme, ‘The World Tonight’, broadcasted on 3rd March 1983.^46^ Leveraging media coverage again, Lord Kennet timed the Bill so its first reading would be the day before it was screened.^47^ Many Peers watched ‘the very disturbing documentary’ and, with some of them learning about the practice for the first time, were horrified by what they saw, agreeing with Lord Kennet that something had to be done to put an end to this ‘barbaric’ practice.^48^

### B. Balancing the need for clear prohibition while respecting medical autonomy

The first point of discussion in the House of Lords was, unsurprisingly, the legal status of female circumcision. The Lord Chancellor delivered a long speech arguing it was already unlawful, as it could be caught under sections 20 or 18 of the Offences Against the Person Act 1861,^49^ a view that was shared by several peers.^50^ However, an explicit prohibition was considered necessary to clear any doubts about the unlawful status of the practice, due to expressivist reasons (sending the unequivocal message that female circumcision would not be tolerated) and substantive justifications (facilitating prosecutions and convictions).^51^ As Marion Roe put it in the House of Commons two years later:

the purpose of the Bill is to make it clear beyond doubt that the practice of female circumcision should be illegal in Britain … it might be argued that to perform such an operation constituted an act of actual bodily harm, [but] there is no precedent detailing this. The degree of abhorrence of these procedures felt by all can leave no room for uncertainty.^52^

The Bill was not presented as a party political matter, attracting support across the aisle.^53^ In fact, given the broad consensus, the media talked about Parliamentary debates being a mere ‘formality’.^54^ Nevertheless, the Bill had an unexpectedly difficult journey—Baroness Trumpington, the then Parliamentary Under-Secretary of State, Department of Health and Social Security, described it a ‘scratchy, bad-tempered affair’.^55^ The main source of contention was the wording and scope of Clause 2, which established a ‘medical exception’ to the criminal prohibition of vulval operations. As the Secretary of State for Social Services had anticipated,^56^ the complexity of legislating in this area lay in that the proscribed conduct of female circumcision is anatomically identical to some vulval surgeries, such as those for cancerous or pre-cancerous treatment, or episiotomy during childbirth.^57^

The first drafting of Clause 2 only allowed for the lawfulness of vulval operations for the ‘physical health of the patient’, as long as this was certified by two medical practitioners from different practices.^58^ Replicating the two-doctor approval of the Abortion Act 1967, a doctor would hence need a second opinion to escape criminal liability upon performing vulval interventions. The medical profession backed the Bill, with the BMA, the Royal College of Midwifes, the Royal College of Nursing (RNC) and the Royal College of Obstetricians and Gynaecologists (RCOG) ‘giv[ing] full support to any measures which will help to eradicate what it regards as a repugnant and unnecessary procedure’.^59^ Nevertheless, the RCOG had reservations, also shared by the Government, about the two-doctor requirement.^60^ They considered it a ‘serious interference with legitimate medical practice’ that would create ‘additional bureaucracy’, cause ‘problems in relation to confidentiality’, and ‘harm the interests of patients by delaying necessary and even life-saving treatment’.^61^ These criticisms were similar to the ones raised by the BMA, RCN, and RCOG regarding the two-doctor requirement within the Abortion Act 1967.^62^ Unlike abortion, however, female circumcision was, at the time, seen as a rare occurrence,^63^ which was another reason why the Government thought second opinions would be unnecessary and burdensome, as they would ‘only impinge upon legitimate medical practice, and will impose a time-consuming burden on doctors and on whoever, at public expense, has to monitor notices’.^64^

In the House of Lords committee stage, an amendment removing the second opinion requirement was agreed to, but then another equally controversial amendment was introduced, expanding the scope of Clause 2 for interventions necessary for *mental* health reasons, as long as this was backed up with the opinion of two consultants in psychiatry.^65^ Lord Kennet, rethinking his initial position, considered that only allowing interventions on physical grounds was ‘too draconian’,^66^ but was concerned about the potential loopholes that the mental health exception could create. As seen above, Shakat used mental health as a justification to operate on his two patients, arguing that female circumcision would alleviate their psychological suffering. Lord Kennet wanted to prevent this kind of scenarios from happening again, so doctors could not (lawfully) operate on ‘depressed African women’ for whom female circumcision was the only way to restore their mental health.^67^ The amendment gave rise to a heated debate, and was strongly criticized for ‘opening the doors wide to abuse’, not providing real safeguards.^68^ Reminiscing ‘the bad old abortion days’, some peers insisted that two opinions offered ‘no protection’, as ‘one chap always asks another chap who he knows would be on the same side’.^69^ Lord Kennet eventually withdrew the amendment, but the seed of discord had already been planted: should the proposed legislation allow for surgeries on mental health grounds and, if so, what provisions, if any, should be put in place to prevent the excessive broadening of this exception?

Attempts to answer this question dominated the rest of the discussions, at the core of which was the tension between protecting the autonomy of the medical profession without rendering the prohibition useless. Lord Rea was appalled by the two-doctor requirement(s), considering them ‘undesirable and even rather insulting by many reputable gynaecologists who have been helping women for decades by operating on this highly personal part of their bodies’.^70^ He sought to protect doctors’ autonomy against ‘legislative scrutiny’,^71^ trusting their judgement in considering when an intervention is medically necessary for ‘cancer, suspected cancer, skin changes which precede cancer and genetic or congenital abnormalities’.^72^ Although he was less sure about the extent to which vulval operations could be ‘taken as legitimate treatments’ for mental health,^73^ he was convinced that doctors would not accept intervening on a woman who is ‘depressed because she has not had this operation which was traditional in her country’ as a medical reason.^74^ That is why he insisted on ‘totally eliminat[ing] these parts of the Bill which are causing problems to the RCOG’.^75^ Several Peers shared Lord Rea’s deferential attitude towards the medical profession, considering it paramount to respect and follow the views of medical experts. For example, Lord Trefgarne, Junior Health Minister at the time, thought that the two-doctor requirement would ‘represent a serious interference with legitimate medical practice’,^76^ and Baroness Masham of Ilton regretted that medical professionals were not present in Parliament to discuss the Bill, since ‘as a lay person, [she could not] argue with the RCOG’.^77^

Nevertheless, not all doctors were entrusted with the same level of respect. During the discussions, there was a continuous distinction drawn between national and foreign medical professionals, where the former were imagined as opposing female circumcision while the latter were seen as more prone to engage with it. As Baroness Hornsby-Smith put it:

I do not think that many English, or should we say Anglo-Saxon, horn doctors are likely to be inclined to pursue this type of operation. It is not unnatural to think that those who have been born and bred in a country where female circumcision was normal practice … are more likely to be the doctors who would perform these operations.^78^

The media contributed to this national vs foreign doctor dichotomy, with news highlighting the non-British (and Global South) origins of doctors who had performed female circumcisions: the BMA magazine *The Doctor* highlighted that Ghatak qualified in Calcutta, and recounted the story of a (British) gynaecologist who had learnt that ‘an Egyptian junior doctor carried out a circumcision on an NHS patient after he refused’.^79^ In a similar vein, Lord Kennet thought that ‘these operations [would] be done by doctors who were born in countries where it was the practice to perform the circumcision of females’, who would also be ‘quite prepared to defend and support a doctor who will carry it out’.^80^ Second opinions were therefore not seen as a sound strategy to police the behaviour of foreign doctors either, as it would be relatively easy for them to find another (foreign) doctor who would sign off the operation. In contrast, British doctors were framed as honourable people who would not want to perform these interventions, and for whom having to seek a second opinion would constitute an unwanted and unjustified interference. The instances of (national) ‘cowboy gynaecologists’ willing to undertake these interventions for ‘private gain’ were considered so small that they did not justify intensifying the monitoring of medical practice.^81^ As Lord Rea wondered: ‘why, in order to prevent what is admittedly a rare occurrence, should [medical professionals’] practice be so scrutinised?’^82^

### C. ‘Custom or Ritual’: The start of a controversial saga

Racialized views carried on with the introduction of the wording ‘custom or ritual’ in the Bill. This section suggests that the origin of this contentious wording can be traced back to a desire to protect the freedom of the medical profession. A general election prevented the original 1983 Bill from reaching the House of Commons, but Lord Kennet reintroduced it in the House of Lords in the summer of 1983, when the direction taken in previous discussions was confirmed. The new Bill allowed for the possibility of performing vulval operations on the grounds of physical *and* mental health, and in either case the approval of only *one* doctor was required. In order to limit the scope of the mental health exception, the Government considered it necessary to clarify that it would exclude operations ‘on the grounds of a desire for it for ritual or customary reasons’, with Lord Glenarthur, Parliamentary Under Secretary of State, introducing an amendment so Clause 2(2) would read as follows:

in determining … whether an operation is necessary for the mental health of a person, no account shall be taken of the effect on that person of any belief on the part of that or any other person that the operation is required as a matter of *custom or ritual*.^83^

The insertion of these last three words stirred a heated debate. Lord Kennet was one of its most vocal critics, condemning it for having ‘a most unhappy racist effect’, since it would allow for ‘white mental health’, but not black mental health, as grounds for the intervention.^84^ The Commission for Racial Equality (now replaced by the Equality and Human Rights Commission) also advised against the clause, which would be ‘indirectly discriminatory in effect’, as doctors would base their judgement over whether performing an operation is acceptable or not on the basis of someone’s origin or descent:

To suggest that some reasons for that state of mind [in assessing mental health] may be acceptable and others, broadly confined to those which might affect persons of African origin or descent, are not, is, in our view, discriminatory and therefore to be avoided.^85^

The amendment was not agreed to, and the Bill passed without it to the House of Commons, where the Government killed it.^86^ Almost a year later, Marion Roe (re)introduced the Bill in the House of Commons with such rejected amendment.^87^ She insisted that the controversial wording should be kept in order to close any loopholes opened by the mental health exception, since ‘we cannot allow female circumcision to occur on the ground that a woman’s health would suffer if she did not conform with the prevailing custom of her community’.^88^ A lengthy discussion about the racialized tone of ‘custom or ritual’ ensued again.^89^ In both Houses, several speeches echoed the Commission’s concerns that the wording was discriminatory and had racialized undertones.^90^ The Government pushed back against these criticisms, insisting that the clause was not in any sense racist, although it did not provide any counterarguments about why it was not, limiting itself to explaining that adding these three words was the only way of honouring the aim of the Bill, while allowing doctors to perform legitimate medical procedures.^91^ Despite the numerous criticisms, the Female Circumcision Act 1985 was passed containing the phrase ‘custom or ritual’, receiving royal assent on 16th of July 1985.

Tracking the two-year journey of the Bill reveals that the means through which Parliament chose to ‘secure the freedom of surgeons’ was through a clause which created a division between Western and non-Western women, allowing the former but not the latter to rely on mental health reasons to have their vulvas operated on.^92^ The first iteration of the Bill contemplated operations for physical reasons only, as long as they counted with the approval of two doctors. In light of the backlash, the two-doctor requirement was removed, and the medical exception was expanded to also cover interventions on the grounds of mental health, with the ‘custom or ritual’ caveat being added to limit the instances where vulval operations would be acceptable. The introduction of this clause was facilitated by the national vs foreign doctor divide, since a central concern was foreign doctors carrying out female circumcision under the auspices of medicine, together with small instances of British doctors who, obeying ‘laws of supply and demand’ ruling on Harley Street, would agree to operate in exchange for money.^93^

The next section explores how the ‘custom or ritual’ wording carried on in subsequent legislation, and the ways in which the changing landscape brought new challenges about how the law should respond to female circumcision. Crucially, no medical professionals—or indeed anyone—were prosecuted under the 1985 Act. Nevertheless, two doctors were struck from the register, one in 1993, for performing female circumcision ‘while knowing it was illegal’,^94^ and another one in 2000, having been secretly videotaped agreeing to ‘circumcise and stitch’ three underage girls.^95^ Dr Abdul Ahmed was contacted by an undercover journalist working on a Channel 4 documentary, who asked him to perform a clitoridectomy on her eight-year-old daughter and her friend’s two daughters.^96^ Ahmed was shown, on tape, agreeing to perform the operations at his home for £50, but he then argued that the tape had been manipulated, and his counsel unsuccessfully told the disciplinary committee that Ahmed was ‘hard of hearing and had believed that he was being asked to circumcise boys’ (in reporting the story, the *BMJ* also emphasized that Ahmed qualified in Calcutta before working in the UK).^97^

## II. The Female Genital Mutilation Act 2003

### A. The aftermath of the 1985 Act and the need for new legislation

By the late 1990s, the absence of criminal prosecutions had become a focal point of political and institutional concern. For example, in 2000, the All-Party Group on Population, Development and Reproductive Health published a report, expressing regret at the failure of enforcement and recommending legislative reform.^98^ In particular, it proposed extending criminal liability extraterritorially, so that UK residents who took girls abroad for vulval cutting could be prosecuted on their return, and replacing the terminology of ‘female circumcision’ for FGM.^99^ This term had been popularized by Fran Hosken, an American journalist who travelled around Africa documenting these interventions, and insisted on using ‘mutilation’ to showcase their horrific nature.^100^ International organizations began to gradually adopt this terminology,^101^ and the UK caught up with the FGM nomenclature in 2003, when Parliament passed the Female Mutilation Act 2003, which repealed and re-enacted the 1985 Act. In addition to the new terminology, the 2003 Act gave extraterritorial effect to FGM-related offences and increased the maximum penalty for FGM from 5 to 14 years imprisonment.^102^ This piece of legislation was introduced as a Private Members Bill in the House of Commons by Anne Clwyd in December 2002. Like the 1985 Act, it counted with cross-party support, and, unlike its predecessor, it had a much easier journey.

A key difference between the 2003 and 1985 discussions lies in the perception and incidence of FGM. In 1985, FGM was seen as a rare occurrence, with legislation being mostly aimed at stopping (foreign) doctors from operating on ‘depressed’ African women.^103^ In 2003, however, the problem seemed to have extended beyond the medical realm, with the BMA estimating that approximately between 3000 and 4000 women underwent FGM every year.^104^ Such increase was linked to a rise in immigration, as Anne Clwyd made clear in her speech: ‘there are 74,000 first-generation African immigrant women in the UK who have undergone FGM, and as many as 7000 girls under 16 within the practising communities who are at risk from FGM’.^105^ Within this new context, the strategy to tackle FGM had to be rethought, and giving extraterritorial effect to FGM-related offences was the chosen path, which was believed would make prosecutions ‘much more likely than in the past’.^106^ Echoing the concerns of the All-Party report, the absence of prosecutions was at the centre of Anne Clwyd’s and many other MPs’ speeches.^107^ It was attributed to several factors, including the closed and isolated nature of FGM practising communities and the young age of victims, but also, and crucially, to the fact that people could circumvent the 1985 Act by travelling abroad.^108^ The discourse tying together a surge in immigration with a rise in FGM incidence continued in the House of Lords, where several peers agreed that the 1985 Act provisions needed to be strengthened to catch those coming back to the UK after engaging with FGM overseas.^109^

The idea of giving extraterritorial effect to the FGM prohibition was uncontroversial, and the debate focused on what its precise extent should be, such as if it should also cover ‘those newly arrived in Britain’ without UK nationality or residency,^110^ and those seeking asylum.^111^ In its final form, Section 4 of the FGM Act 2003 extended its jurisdiction to acts ‘done outside the UK by a UK national or a permanent resident’.^112^

Despite the optimism about how these reforms would boost prosecutions, the first ever prosecution for FGM was not brought until 2015,^113^ and the first prosecution of an FGM-related offence carried out abroad had to wait until 2023. Amina Noor, a Somali-born British citizen, was convicted for assisting a Kenyan woman to perform FGM on a three-year-old British girl during a trip to Kenya in 2006.^114^ The police investigation started in 2018, when the victim, then aged 16, told one of her teachers about having undergone FGM.^115^ Medical examinations confirmed that she had had her clitoris removed.^116^ In 2006, Noor took the then three-year-old girl to Kenya and brought her to a ‘clinic place’, where she had the intervention.^117^ Her defence was that she did not think that the operation would be so invasive, but that the girl would ‘simply be “touched”’, and that she did not assist or encourage the person doing the procedure, not even going inside the place where it was carried out.^118^ Nevertheless, even the ‘smallest’ cut amounts to an offence under Section 1 of the 2003 Act, and evidence was found that she did go inside the ‘clinic place’, which turned out to be a private house with no doctors.^119^ She was convicted under Section 3 of the FGM Act 2003—assisting a non-UK person to mutilate overseas a girl’s genitalia—and sentenced to seven years’ imprisonment. She unsuccessfully appealed her sentence, with the Court of Appeal confirming that the judge ‘conducted this complex sentencing exercise in exemplary fashion’,^120^ and that the sentence ‘was not manifestly excessive’.^121^

### B. ‘Custom or Ritual’: Continuing the saga

As Section 1 showed, the main aspect that hindered the passing of the 1985 Act was the scope and terms of the medical exception, which eventually allowed for the performance of interventions necessary for the patient’s physical and mental health, with the controversial caveat that ‘no account’ could be taken, for assessing the latter, of whether the patient considered that the operation was required as a ‘matter of custom or ritual’.^122^ The 2003 Act incorporated the same caveat in Section 1(5), establishing that ‘for the purpose of determining whether an operation is necessary for the mental health of a girl it is immaterial whether she or any other person believes that the operation is required as a matter of custom or ritual’.

The clause that had been the source of so much disagreement 20 years prior was less contentious in 2003, when there was no ‘meaningful debate’ about its terms and extent.^123^ At the committee stage, David Wilshire proposed an amendment to remove the justification on the grounds of mental health, bringing back the loophole concerns rehearsed during the 1985 Act discussions.^124^ However, he withdrew it after Anne Clwyd and several MPs defended the necessity of a mental health exception.^125^ He was particularly persuaded by Dr Tonge’s speech, who drew on her experience as a medical professional to contend that ‘there are genuine mental health reasons for carrying out operations’.^126^ Showing deference for the medical profession in a tone that reminded of some interactions from the 1985 Act discussions, he ‘unhesitatingly accept[ed] that the clause was necessary if a medical practitioner said so’.^127^ Some members also brought back criticisms about the double standards set by the ‘custom or ritual’ wording,^128^ with Sandra Gidley going as far as to say that ‘no distinction’ should be drawn between the two practices, as vulval cosmetic surgery should also be considered illegal under the 2003 Act: ‘we should not make any exceptions for white women expressing a choice for fashion reasons, when we are stopping black women … from having surgery’.^129^

RAINBO made the same point, criticizing that only ‘white girls’ are allowed to have their vulvas operated on, when both white and black girls are ‘responding to the demands of the “cultural” aesthetics of their generation’.^130^ However, in light of the Act, only those who belong to communities where FGM is practiced are seen as having culturally determined beliefs, following ‘rituals or customs’. In contrast, those who pursue Western beauty ideals are not culturally oppressed, but bodily dissatisfied, for which surgery constitutes a legitimate treatment to restore their mental health.^131^ Challenging this inconsistency, RAINBO recommended that the prohibition should apply to all underage minors, regardless of their race or ethnic origin, but *not* to ‘consenting adults regardless of their reasons of their request that is whether cosmetic or customary’.^132^ Adrienne Shahvisi makes a similar suggestion, contending that non-therapeutic forms of genital cutting for all children should be prohibited, and more caution should be exercised when restricting the choices of adult women.^133^ Also appealing to consistency but reaching a different conclusion, Clare Chambers, like Gidley, argues that there are good reasons for prohibiting both vulval cosmetic surgery and FGM on adults, as they are both ‘seriously risky or harmful and strongly connected to sex inequality’.^134^ The Act currently prohibits FGM both for underage and adult women, all of whom are referred to as ‘girls’, with Section 6(1) explaining that ‘girl includes woman’. The use of the term ‘girl’ has been harshly criticized for its infantilizing undertones, as though women of colour were ‘incapable of consenting to surgery on their genitalia’,^135^ presuming that they lack agency and capacity for deliberate choice.^136^

If the goal is to protect people from harm, Moira Dustin and Anne Philips point out that the prohibition should be framed in different terms: rather than distinguishing between ‘cultural’ and ‘cosmetic’ cuts, it would be more coherent to ban ‘more intrusive forms of cosmetic surgery but permit less intrusive forms of circumcision’ for adult women.^137^ Interestingly, Lord Kennet made the same suggestion in 1985: the prohibition should cover ‘grosser procedures’, not the ‘smallest trimmings or corrections’, since, if ‘minor’ vulval operations are ‘all right for a white girl who is depressed about her appearance, then [they] should be all right for a black girl who is depressed as a matter of custom’.^138^ Nevertheless, none of these suggestions was ever adopted, and the 2003 Act remains to reify the divide between black and white mental health, differentiating between cultural and cosmetic cutting (the Act itself does not mention cosmetic surgery, but Explanatory Notes use it as an explicit example of operations necessary for mental health^139^).^140^

The fact that the law in 2003 did not change does not mean that concerns raised in academic circles and the 1985 Act Parliamentary discussions were unfounded. In 2014, the House of Commons Affairs Committee recommended a re-examination ‘of the extent to which there is a double standard’,^141^ concluding in 2016 that vulval cosmetic surgery should be prohibited as well, since ‘we cannot tell communities in Sierra Leone and Somalia to stop a practice which is freely permitted in Harley Street’.^142^ The Nuffield Council on Bioethics, the RCS, the RCOG, and the BMA have complained about the uncertainty current legislation generates.^143^ Two examples are illustrative of the complex overlap between FGM and vulval cosmetic surgeries. First, the case of Sureshkumar Pandya, a London GP whose fitness to practice was challenged in 2012. He had performed a labiaplasty on a 33-year-old woman who felt uncomfortable because she thought her vulva was ‘ugly’ and her ‘labia felt too big’.^144^ The patient was unhappy with the surgery and sought a second opinion from another GP because the procedure was not ‘as expected’ and she felt she was not told ‘the whole truth … wondering if she had been circumcised’.^145^ Pandya admitted that the surgery did not go as planned, but denied having had any intention to perform FGM or to remove the patient’s inner labia, explaining that the result of surgery was the materialization of a risk present even where all ‘due care, vigilance and manual dexterity’ is exercised.^146^ He was eventually considered fit to practice, although he admitted that he should have kept accurate records of the preoperative discussions with the patient, which is now considered good practice in accordance with the RCOG’s guidance,^147^ and a relevant factor when considering whether prosecution would be in the public interest under Crown Prosecution Service (CPS) Guidance relating to FGM (see further discussion below).

Second, in 2011, a psychiatrist and a surgeon were involved in the performance of a clitoridectomy, also to a 33-year-old woman, who reported suffering from a life-long aversion to her genitals, even though there were no diagnosable problems in her clitoris or labia.^148^ She had already undergone a labiaplasty the year before and now requested to have her clitoris removed. Despite her persistent discontent about her vulva, she was considered not to tick the boxes of any psychiatric disorder, and the psychiatrist in that case concluded that ‘it was safe to proceed’, considering that the surgery would have ‘psychosocial’ benefits to the patient’.^149^ After her clitoridectomy, the patient was reported to be ‘very grateful and satisfied … [and] pleased with the improvement in her sexual life’.^150^ The doctors published the case in *Archives of Sexual Behaviour*, calling for further research on the psychological implications of vulval cosmetic surgery.^151^ After reading about it, Professor and obstetrician Susan Bewley referred the case to the CPS, as she believed that the surgical procedure amounted to FGM.^152^ Three years of investigation ensued, but eventually no charges were brought.^153^ The two medical professionals who were involved in the surgery always maintained that what they did was not comparable to the ‘abhorrent practice’ of FGM, which is ‘conducted on girls against their consent motivated by a desire to control female sexuality’.^154^ In contrast, the operation they performed was ‘for adult women with capacity to consent and motivated by a desire to improve their appearance and sexuality’.^155^

The RCOG warns in its 2015 guidance that ‘[vulval cosmetic surgery] may be prohibited unless it is necessary for the patient’s physical or mental health’.^156^ In order to ensure and demonstrate compliance with the law, the RCOG considers it good medical practice that written records of the mental or physical reasons why the procedure is deemed necessary are kept, as well as patient consent forms.^157^ Moreover, the CPS issued guidance shedding some more light on when surgeons who perform vulval cosmetic surgery might be more likely to face prosecution. In order for the medical exception in Section 1(2) to apply, surgery must have, the CPS insists, a ‘therapeutic element’.^158^ It might be of a psychological nature, and can thus be present when the surgery ‘has a cosmetic element, if there is some evidence available of medical reason for the procedure’. However, such defence will not apply ‘where the surgery is purely to alter the appearance of the genitals’.^159^ If the health necessity fails, the CPS must consider whether prosecution would be in the public interest, setting out a list of factors in favour and against prosecution, such as severity and invasiveness of the procedure, harm caused to the victim, risk of future harm, medical qualification of the person performing the procedure, documented evidence of the informed consent process, evidence of marketing or advertising, with inaccurate claims, or evidence of financial benefit.^160^

Only one of the prosecutions brought so far has been against a medical professional, Danuson Dharmasena. He sutured his patient’s labia following an incision made to enable the delivery of her baby, as she had been previously infibulated.^161^ The patient did not make a complaint about FGM, and in fact gave evidence for the defendant on trial—it was a midwife working with Dharmasena who referred the case to the police.^162^ A year and a half after the incident occurred, Alison Saunders, the Director of Public Prosecutions (DPP), announced that Dharmasena was going to be the first person charged for FGM in the UK. The announcement of this landmark prosecution was made three days before Saunders was scheduled to give evidence to the House of Commons Affairs Committee about the lack of prosecutions for FGM (see below for further discussion).^163^ Dharmasena was eventually acquitted, as the jury returned with a verdict of not guilty after a 30 minute deliberation.^164^ His prosecution was harshly criticized by the medical profession, with a group of consultant obstetricians and gynaecologists writing a letter to *The Guardian* where they explained that ‘there is the world of difference between FGM and repairing cuts necessary to allow a baby’s delivery’.^165^

Within the 18 years that lapsed between the 1985 and the 2003 Act, FGM came to be constructed as a widespread problem within migrant communities, and this construction displaced earlier debates about medical discretion and exception. Within this context, making the criminal law more effective, particularly through giving it extraterritorial effect, rather than reflecting about the scope and terms in which medical professionals may perform vulval interventions, became the central topics of 2003 law reform. The ‘custom or ritual’ wording, while receiving some pushback and sparking some debate, was not a problem that challenged the passing of the Bill like it did in the 1980s. Nevertheless, the distinction between vulval cosmetic surgery and FGM is far from settled. CPS guidance clears some of the fuzziness, but no test case has yet been brought where a surgeon is accused of performing FGM under the auspices of cosmetic surgery. The closer we have is the case of Dharmasena, although the intervention he performed was justified on the grounds of physical health, and the 2003 Act explicitly contemplates the scenario where the obstetrician found himself in, with Section 1(2)(b) clarifying that no offence is committed if an approved person performs ‘a surgical operation on a girl who is in any stage of labour, or has just given birth, for purposes connected to the labour or birth’.

The next section examines the last chapter of legislative activity with regard to FGM, which took place when the Serious Crime Act 2015 amended the 2003 Act. 2015 reforms intensify criminalization and reconfigure the role of medical professionals, who have been allocated new responsibilities of reporting FGM.

## III. The Serious Crime Act 2015

### A. The aftermath of the 2003 Act and the need further legal change

As explained above, it took more than 10 years since the passing of the 2003 Act, and almost 30 years since FGM was explicitly criminalized, for the CPS to bring the first prosecution for FGM. The lack of prosecutions was not due to a lack of interest: before charging Dharmasena, the CPS had reviewed three cases where it had decided not to go forward due to insufficient evidence.^166^ A central barrier for prosecution were the low reporting rates of FGM, since ‘many victims may be too young and vulnerable or too afraid to report offences to the police’, and ‘many not wish to report their parents’.^167^ As a result, the police faced considerable evidential difficulties when conducting an investigation, including silence from the community and challenges in obtaining reliable and admissible evidence, particularly if the cutting had taken place abroad.^168^ Seeking to address this, the CPS created specific guidance for prosecutors regarding FGM, organized two round tables with police, third sector organizations and professional bodies, and published a new action plan in 2013 which encouraged early consultation and collaboration between the CPS and police to ‘ensure that all avenues of evidence are explored and that the correct charged is identified’.^169^

These measures were, however, not enough and, in February 2014, Saunders wrote to Ministers in the Home Office, Ministry of Justice and Department of Health proposing different ways in which ‘the criminal law could be strengthened’.^170^ Her suggestions included making explicit that ‘re-infibulation’ fell under the scope of the 2003 Act, broadening the extraterritorial scope of the Act, placing a positive duty on parents to protect their children from FGM, and creating a new statutory duty on health and education professionals to report FGM.^171^ These suggestions featured in the Queen’s Speech in June 2014,^172^ and were seen favourably by the House of Commons Affairs Committee one month later.^173^ That same month, the then Home Secretary Theresa May hosted the Girl Summit in London, where she announced an ‘unprecedented package of measures to tackle FGM’.^174^

With a considerable amount of public attention and momentum, the Government introduced the Serious Crime Bill which, among other reforms, amended the 2003 Act to introduce some of the proposed changes. Like in 2003 and 1985, FGM was ‘not a political party matter’ but something Parliament was ‘united in tackling’,^175^ and achieving successful prosecutions for FGM was deemed the best method to condemn the practice and protect those at risk.^176^ The DPP’s and Committee’s recommendation of explicitly including ‘re-infibulation’ in the definition of FGM was not taken forward. The Government’s ‘firm view’ was that re-infibulation was already covered by Section 1(1), and clarification would be better achieved instead by communicating the law more to practising communities and relevant stakeholders, particularly medical professionals.^177^

The Bill originally introduced four amendments to the 2003 Act. First, extraterritorial jurisdiction was expanded to those ‘habitually resident’ in the UK. The Parliament Under-Secretary of State for the Home Department, Karen Bradley, explained how what was considered a ‘ground-breaking provision’ in 2003 was ‘less unusual’ in 2015, and that the law needed to ‘go further’.^178^ FORWARD, an African women-led organization working to end violence against women and girls, and the Metropolitan Police agreed that ‘catching’ only those who have permanent residence in the UK left ‘a significant loophole’ which hampered the ability to investigate and prosecute.^179^ Second, replicating the protection of victims of sexual offences, the Bill introduced lifelong anonymity for victims of FGM.^180^ Third, focusing on prevention, the Bill created the new legal remedy of FGM Protection Orders, a hybrid criminal-civil law measure modelled after Forced Marriage Protection Orders, to protect girls and women at risk of FGM. Fourth, it added the new offence of failing to protect a girl from the risk of FGM, ‘shifting responsibility’ to parents and carers of a girl under 16 ‘to make them accountable’ for stopping their child from being subjected to FGM.^181^

### B. Mandatory reporting and recording

In December 2014, the Home Office issued a consultation seeking views on whether and how to introduce mandatory reporting for FGM.^182^ The DPP,^183^ and House of Commons Affairs Committee had concluded this was a necessary step, given that one of the main reasons for the very few police investigations was that victims do not report, and that health, educational and social care professionals do not issue referrals to the police either.^184^ As a result of the consultation, the Government tabled amendments to the Serious Crime Bill in February 2015 to create a new duty to report.^185^ With this change, regulated professionals have to report to the police where, in the course of their professional duties, are ‘informed by a girl under 18 that an act of FGM has been carried out on her’; or ‘observe physical signs which appear to show that an act of FGM has been carried out on a girl under 18 and they have no reason to believe that the act was necessary for the girl’s physical or mental health or for purposes connected with labour or birth’.^186^

Mandatory reporting was made a statutory duty despite clear opposition from health and social care professionals.^187^ Members from the Royal College of General Practitioners, Royal College of Midwifes and RCOG gave evidence contending that ‘blaming health professionals for a failure to prosecute is not putting the blame in the right place’.^188^ Such duty would generate extra time constraints and undermine trust in the system, as doctors would ‘cease to be seen as independent providers of medical care and [would] be viewed … as part of the criminal justice system’.^189^ It would risk deterring those subjected to FGM from seeking medical assistance,^190^ who would be put in a position of greater vulnerability and stigmatization.^191^ At the core of their critique was that a ‘blanket mandatory obligation’ would not take into account the individual circumstances of each patient, for whom reporting might not always be beneficial,^192^ contrasting it with GMC guidance on when doctors should report to the police.^193^ Moreover, reporting every case of FGM could potentially be too burdensome for the police, whose ability to investigate, now hindered by under-reporting, could be adversely impacted by over-reporting, potentially struggling in prioritizing case-loads.^194^

Some members of Parliament accused doctors of ‘hiding behind confidentiality’ to dodge their role in identifying and referring cases of FGM to the police.^195^ This comment, read together with the introduction of mandatory reporting, signals a shift from the 1980s. Back then, the stance of the medical profession led to the removal of the initial two-doctor requirement and the introduction of the controversial ‘ritual or custom’ clause. In contrast, Parliament in 2015 passed a measure which most relevant colleges were against (a notable exception was the RCN, which showed support for the introduction of mandatory reporting).^196^ At the same time, the introduction of this duty also reflects a change in the perception of the role of the medical profession. No longer just seen just as a body of experts whose autonomy needs protecting, they have become inserted in the criminal justice system, which needs to be more effective in investigating, prosecuting, and convicting those involved in FGM. Being scooped by the strategy of criminalization, the medical profession is not just involved in the regulation of FGM through delimiting the periphery of the criminal prohibition (with the ever-controversial debate of cosmetic vs cultural cuts), but is now a key facilitator in the deployment of coerciveness.

In fact, obligations of the medical profession with regard to FGM do not end with their duty to report, as there is also a duty to record in place. Calls for gathering more data about FGM featured in 2003 Parliamentary discussions,^197^ and the Health and Social Care Act 2012 introduced an obligation to record patient data, for which the FGM Enhanced Dataset was created and implemented in June 2015.^198^ Since then, all acute NHS Trusts and GPs must submit details of all cases of FGM that come to their knowledge to the Health and Social Care Information Centre. The data includes type of FGM, age of the patient when FGM was performed, family history of FGM, whether the woman has any daughters and whether she is or becomes pregnant.^199^ The data is not anonymized at the point of submission although it is anonymized for publication of quarterly reports.^200^ The introduction of such duty to record was also met with reluctance, with several senior clinicians expressing their concerns about criminalization-led governance and the strain placed on professional–patient trust in a letter to the *British Medical Journal*:^201^ mandatory recording ‘compromises confidence closer to breaking point’, has ‘no evidence of benefit’, ‘wast[es] precious clinical time’ and ‘profoundly damages trust in health professionals, who will either collude or ignore the imperative’.^202^ In 2024, NHS England Digital opened a consultation to review whether the FGM Enhanced Dataset was still ‘fit for purpose’, and whether information could be provided in a ‘least burdensome and sensitive manner’.^203^

## IV. CONCLUSION

This article has traced the ways in which criminalization, race, and medical authority intersect across the three legislative phases of vulval cutting, showing how the criminal justice system has been stabilized as a core, and increasingly prominent, feature in the UK’s approach to tackle FGM. The 1985 Act was passed with the main goal of making clear to medical professionals that female circumcision was unlawful. The ‘custom or ritual’ provision was introduced so doctors would not justify this intervention on the basis of mental health while also safeguarding their freedom in performing vulval operations without needing further approval. This was the origin of the much-disputed divide between vulval cosmetic surgery and female circumcision/FGM, which not only establishes a hypocritical double standard between legitimate and illegitimate body image concerns, but also, as the investigations and CPS guidelines showcase, fails to draw a clear line between lawful and unlawful cuts.

One might wonder whether concerns about doctors’ freedom, so important in shaping the 1985 Act, still stand to justify the ‘ritual or custom’ wording. As seen in the last section, Parliamentary debates in 2015 show a less deferential attitude towards the medical profession, culminating in the introduction of an obligation to report FGM to the police. In the last 40 years, the regulatory debate’s focus has moved from the role of doctors in defining the limits of the criminal ban to how to integrate the medical profession in enforcing the prohibition. In turn, FGM has evolved from being seen as an intervention contained within the clinic which (non-Western) doctors must refrain from performing, to a wider phenomenon within migrant communities requiring an integral approach to facilitate the identification, investigation, prosecution, and conviction of those involved in it. Yet, revisiting the history of FGM reveals that this is not a tale of success. Despite its constant expansion since 1985, and notwithstanding the timid upwards prosecutorial trend in recent years, the criminal justice system set in place for vulval cutting has not borne fruit as expected: the contours of the prohibition are confusing, the number of convictions is very small, and the expressivist power of the criminal law is weak amongst those targeted by it, as the degree of awareness about FGM being a criminal offence among practicing communities remains low.^204^

Analysing the historical shifts in the regulation of vulval cutting illuminates the past in order to ‘disrupt the certainties of the present’.^205^ The strategy of criminalization (and the evolving role of medical professionals within it) is not natural or obvious, but this article has shown that it has a particular history, being the result of constantly changing negotiations with (and perceptions of) the medical profession. In doing so, this article has exposed how the most challenging ‘features’ of the existing framework (the unclear lawfulness of vulval surgeries on the grounds of mental health, the low rate of prosecutions and convictions, the challenges with doctors’ duty to report) came into being, and the role that racialized ideas of medical legitimacy and mental health played in this process. In doing so, this article provides the groundwork for articulating a criticism of the regulation of vulval cutting which goes beyond exposing its problematic symptoms, moving towards interrogating its foundations and logics. Looking ahead, attention must be paid to the implications of placing criminalization at the centre of legislative efforts, examining how those vulnerable to vulval cutting might be alienated by this strategy, assessing the impact of blurring medical care with criminal surveillance, and starting to deconstruct the conflation between using the criminal law and protecting people from harm.^206^

## Footnotes

1 Prohibition of Female Circumcision Act 1985, s1(1)(a).

2 ibid s2(1).

3 In Scotland, the practice is illegal under the Prohibition of Female Mutilation (Scotland) Act 2005, which was amended by the Female Genital Mutilation (Protection and Guidance) (Scotland) Act 2020.

4 Sally Sheldon and others, ‘The Abortion Act (1967): A Biography’ (2019) 39 Legal Studies 18, 19.

5 ibid.

6 Janice Boddy, ‘Legislating Against Culture: Efforts to End Pharaonic Circumcision in the Anglo-Egyptian Sudan’ (2008) 33 Suomen Antropologi: Journal of the Finnish Anthropological Society 17; Katherine Angela Luongo, ‘The Clitoridectomy Controversy in Kenya: The “Woman’s Affair” That Wasn’t’ (2000) 28 Ufahamu: A Journal of African Studies 105.

7 Ruth Gaffney-Rhys, ‘From the Offences Against the Person Act 1861 to the Serious Crime Act 2015 - the Development of the Law Relating to Female Genital Mutilation in England and Wales’ (2017) 39 Journal of Social Welfare and Family Law 417; Charlotte Proudman, *Female Genital Mutilation. When Culture and Law Clash* (Oxford University Press 2022) chap 2.

8 K Hayter, ‘Female Circumcision–Is There a Legal Solution?’ (1984) 6 The Journal of Social Welfare Law 323; Elise A Sochart, ‘Agenda Setting, the Role of Groups and the Legislative Process: The Prohibition of Female Circumcision in Birtain’ (1988) 9 Parliamentary Affairs 508; Ruth Gaffney-Rhys, ‘Recent Developments in the Law Relating to Female Genital Mutilation’ (2016) 28 Child and Family Law Quarterly 87.

9 Yusuf Malik and others, ‘Mandatory Reporting of Female Genital Mutilation in Children in the UK’ (2018) 26 British Journal of Midwifery 377; Joseph Home and others, ‘A Review of the Law Surrounding Female Genital Mutilation Protection Orders’ (2020) 28 British Journal of Midwifery 418.

10 Ruari D Mcalister, ‘A Dangerous Muddying of the Waters?’ (2016) 24 Medical Law Review 259; Brian D Earp, Jennifer Hendry and Michael Thomson, ‘Reason and Paradox in Medical and Family Law: Shaping Children’s Bodies’ (2017) 25 Medical Law Review 604.

11 Charlotte Proudman and Nkumbe Ekaney, ‘Reviewing the Law on Travel Bans in FGM Cases’ (2019) 49 Family Law 1305.

12 Marge Berer, ‘Prosecution of Female Genital Mutilation in the United Kingdom: Injustice at the Intersection of Good Public Health Intentions and the Criminal Law’ (2019) 19 Medical Law International 258; Hartej Saund-Matharu and Joe Home, ‘*R v Noor*: A Landmark Case in Female Genital Mutilation Prosecution.’ (2024) 32 British Journal of Midwifery 440.

13 IR Gunning, ‘Arrogant Perception: Traveling and Multicultural Femininism: The Case of Female Genital Surgeries’ (1992) 23 Columbia Humans Rights Law Review 189; Richard A Shweder, ‘What about “Female Genital Mutilation”? And Why Understanding Culture Matters in the First Place’ (2000) 129 Daedalus 209; Obioma Nnaemeka (ed), *Female Circumcision and the Politics of Knowledge: African Women in Imperialist Discourses* (Praeger 2005).

14 Nawal El Saadawi, *The Hidden Face of Eve* (Zed Books 1980) 40; Fuambai Ahmadu, ‘Rites and Wrongs: An Insider/Outside Reflects on Power and Excision’ in Bettina Shell-Duncan and Ylva Hernlund (eds), *Female ‘Circumcision’ in Africa: Culture, Controversy, and Change* (Lynne Rienner Publishers 2000).

15 Katherine Brennan, ‘The Influence of Cultural Relativism on International Human Rights Law: Female Circumcision as a Case Study’ (1989) 7 Law and Inequality 367; Bronwyn Winter, Denise Thompson and Sheila Jeffreys, ‘The UN Approach to Harmful Traditional Practices’ (2002) 4 International Feminist Journal of Politics 72.

16 Moira Dustin, ‘Female Genital Mutilation/Cutting in the UK: Challenging the Inconsistencies’ (2010) 17 European Journal of Women’s Studies 7; B Kelly and C Foster, ‘Should Female Genital Cosmetic Surgery and Genital Piercing Be Regarded Ethically and Legally as Female Genital Mutilation?’ (2012) 119 BJOG: An International Journal of Obstetrics and Gynaecology 389; Lisa Avalos, ‘Female Genital Mutilation and Designer Vaginas in Britain: Crafting an Effective Legal and Policy Framework’ (2014) 48 Vanderbilt Journal of Transnational Law 621; Sarah M Creighton and Lih-Mei Liao (eds), *Female Genital Cosmetic Surgery. Solution to What Problem?* (Cambridge University Press 2019).

17 World Health Organization (WHO), ‘Eliminating Female Genital Mutilation. An Interagency Statement’ (2008) 10–13.

18 Women and Equalities Committee, *Female Genital Mutilation* (HC 2024-2025, 714-VII) para 121 (Conclusion).

19 ibid para 57 (Conclusion).

20 Efua Dorkenoo and Scilla Elworthy, ‘Female Genital Mutilation: Proposals for Change’ (Minority Rights Group 1992); ‘Rainbo’ <https://www.rainbo.org/> accessed 11 November 2025; ‘FORWARD’ <https://www.forwarduk.org.uk./> accessed 11 November 2025.

21 For further discussion, see Moira Dustin and Anne Phillips, ‘Whose Agenda Is It?: Abuses of Women and Abuses of “culture” in Britain’ (2008) 8 Ethnicities 405, 415–6.

22 Sandra Leville, ‘Doctor Found Not Guilty of FGM on Patient at London Hospital’ *The Guardian* (4 February 2015); Steven Morris, ‘UK FGM Trial: Father Says Failed Case Put Intolerable Pressure on Him’ *The Guardian* (23 February 2018).

23 Sarh Marsh, ‘Mother Jailed for 11 Years in First British FGM Conviction’ *The Guardian* (8 March 2019).

24 This case features in the analysis of Section II as it is useful to contextualize and assess the extraterritorial scope of the prohibition brought by the 2003 Act and expanded by the 2015 reforms.

25 Crown Prosecution Service, ‘Jail for Legal First Female Genital Mutilation Conspiracy (3 October 2024) <https://www.cps.gov.uk/east-midlands/news/jail-legal-first-female-genital-mutilation-conspiracy> accessed 7 August 2025.

26 For an examination of the challenges in defining and deploying racialisation as an analytic concept, see Karim Murji, *Racism, Policy and Politics* (Policy Press 2017) Ch 2.

27 Sarah Ahmed, ‘Racialized Bodies’ in Mary Evans and Ellie Lee (eds) *Real Bodies* (Macmillan Education 2002) 46.

28 WHO (n 17) 33.

29 Women and Equalities Committee (n 18) para 74.

30 Shyam Shatia, ‘Female Circumcision: Harley St Performs “Barbaric” Operation’ *The Observer* (10 October 1982).

31 ibid.

32 Mary Bishop, ‘GMC “Powerless” on Female Circumcision’ *The Hospital Doctor* (21 October 1982).

33 ibid.

34 Shatia (n 30).

35 Bishop (n 32).

36 ibid.

37 ibid.

38 HL Deb 9 November 1982 WA, col 139.

39 HL Deb 23 June 1982 WA, col 117.

40 HL Deb 28 June 1982 WA, col 139.

41 Shyam Shatia, ‘Outlaw This Operation’ *The Observer* (17 October 1982).

42 ‘Operation on Girls “Illegal”’ *The Observer* (19 December 1982).

43 ibid.

44 HL Deb 21 April 1983, vol 441, col 675.

45 ibid col 674.

46 Shyam Shatia, ‘New Evidence on Female Mutilation’ *The Observer* (27 February 1983).

47 Sochart (n 8) 512.

48 HL Deb 21 April 1983, vol 441, col 881 (Lord Rea), col 683 (Baroness Cox), col 695 (Baroness Jeger), col 688 (Baroness Vickers).

49 ibid col 677.

50 Nevertheless, the Lord Chancellor also made clear that he was not making this point to argue against the Bill, as he insisted that he was ‘simply stating what [he] believe[d] to be the criminal law at the present time’. See ibid col 677.

51 ibid col 680 (Lord Rea), col 694 (Baroness Jeger); Hayter (n 8) 330–3.

52 HC Deb 19 April 1985, vol 77, col 58. Mr Kennet Clarke, Minister of Health at the time, made the same point, see col 587.

53 HL Deb 21 April 1983, vol 441, col 674.

54 ‘Bringing an End to Mutilation’ *New Statesman* (11 November 1983).

55 HL Deb 12 September 2003, vol 652, col 664.

56 ‘Operation on Girls “Illegal”’ (n 42).

57 HL Deb 21 April 1983, vol 441, col 675.

58 ibid.

59 ibid.

60 ibid col 676.

61 ibid col 679 (Lord Trefargne). Baroness Masham raised this point as well in col 686.

62 House of Common, Committee, *Scientific Issues Relating to Abortion* (2006-2007), para 88.

63 HL Deb 21 April 1983, vol 441, col 681 (Lord Rea), col 695 (Baroness Jeger), col 689 (Baroness Ewart-Biggs).

64 ibid col 679.

65 HL Deb 10 May 1983, vol 442, col 451.

66 ibid col 452.

67 ibid.

68 ibid col 452 (Baroness Jeger), col 454 (Lord Hatch).

69 ibid col 453.

70 HL Deb 13 May 1983, vol 442, col 725.

71 ibid.

72 HL Deb 21 April 1983, vol 441, col 686 (Baroness Misham of Ilton).

73 HL Deb 13 May 1983, vol 442, col 729 (Lord Rea).

74 ibid col 725 (Lord Rea).

75 ibid.

76 HL Deb 21 April 1983, vol 441, col 678.

77 HL Deb 15 May 1985, vol 463, col 1244.

78 HL Deb, 10 May 1983, vol 442, col 444.

79 Bishop (n 32).

80 HL Deb 13 May 1983, vol 442, col 727. Female circumcision has been documented in several countries, mostly in Africa and Asia, including Burkina Faso, Cameroon, Egypt, Ethiopia, Gambia, Ghana, Kenya, Mali, Nigeria, Senegoa, Somalia, Uganda, and Malaysia. For more information regarding the prevalence of the practice, see WHO (n 17) Annex 3.

81 ibid col 725.

82 ibid col 725.

83 HL Deb 23 January 1984, vol 447, col 74 (emphasis my own).

84 ibid col 78.

85 ibid quoted from col 78.

86 Colin Brown, ‘Government Blocks Bill on Female Circumcision’ *The Guardian* (28 April 1984).

87 Sochart (n 8) 520.

88 HC Deb 19 April 1985, vol 77, col 584.

89 HL Deb 3 June 1985, vol 464, cols 572–591; HL Deb 18 June 1985 vol 465, cols 207-24; HL Deb 2 July 1985, vol 465, cols 1135–1148.

90 HL Deb 15 May 1985, vol 463, col 1266; HC Deb 19 April 1985, vol 77, col 585.

91 HL Deb 2 July 1985, vol 465, col 1142.

92 HL Deb 23 January 1984, vol 447, col 78.

93 Bishop (n 32).

94 British Medical Association, ‘Female Genital Mutilation: Caring for Patients and Child Protection’ (1996) 4.

95 Sarah Boseley and Health Editor, ‘Doctor Struck off over Genital Surgery’ *The Guardian* (20 December 2000).

96 Owen Dyer, ‘GP Struck Off for Agreeing to Perform Female Circumcision’ (322) 322 British Medical Journal 9, 9.

97 ibid.

98 All-Party Parliamentary Group on Population, Development and Reproductive Health, ‘Report of the Parliamentary Hearings on Female Genital Mutilation Held on 23 and 24 May 2000. November 2000’ (2000) 4.

99 ibid.

100 Fran P Hosken, *The Hosken Report: Genital and Sexual Mutilation of Females* (Women’s International Network News 1994).

101 Fourth World Conference on Women, ‘Beijing Declaration and Platform for Action’ (1995) para 39.

102 Explanatory Notes to the Female Genital Mutilation Act 2003, para 5.

103 ‘Bringing an End to Mutilation’ (n 54).

104 HC Deb 21 March 2003, vol 401, col 1197.

105 ibid col 1189.

106 Female Genital Mutilation Bill 25 June 2003, col 006.

107 HC Deb 21 March 2003, vol 401, col 1191 (Anne Clwyd), col 1194 (Marion Roe), col 1197 (Chris McCafferty), col 1200 (Sandra Gidley), col 1204 (Cheryl Gillan).

108 ibid col 1191.

109 HL Deb 12 September 2003, vol 652, col 641 (Baroness Gould), col 642 (Barones Finlay).

110 Adwoa Kwateng-kluvitse, Director of FORWARD, personal communication, 17 March 2003, quoted in House of Commons Research Paper 03/24, The Female Genital Mutilation Bill, Bill 21 of 2002–2003.

111 Female Genital Mutilation Bill 25 June 2003, col 10.

112 Explanatory Notes to the Female Genital Mutilation Act 2003, para 11.

113 See Section II.B for further discussion.

114
*R v Amina Noor (Sentencing Remarks)*, Central Criminal Court, 16 February 2024.

115
*R v Noor* [2024] EWCA Crim 714 [17].

116 ibid.

117 ibid [15].

118 ibid [18].

119
*R v Amina Noor (Sentencing Remarks)* (n 114) [25].

120
*R v Noor* (n 115) [73].

121 ibid [76].

122 Prohibition of Female Circumcision Act 1985, s2(2).

123 Proudman (n 7) 66.

124 Female Genital Mutilation Bill 25 June 2003, col 1556.

125 HC Deb 11 July 2003, vol 408, col 1557.

126 ibid col 1157.

127 ibid col 1158.

128 HC Deb 12 September 2003, vol 652, col 638.

129 HC Deb 21 March 2003, vol 40, col 1200.

130 RAINBO, comments on Female Genital Mutilation Bill, personal communication, quoted in House of Commons Research Paper 03/24 (n 110) 36.

131 Dustin (n 16) 16.

132 RAINBO, comments on Female Genital Mutilation Bill, personal communication, quoted in House of Commons Research Paper 03/24 (n 110) 36.

133 Arianne Shahvisi, ‘Why UK Doctors Should Be Troubled About UK Legislation’ (2017) 12 Clinical Ethics 102, 106.

134 Clare Chambers, ‘Medicalised Genital Cutting and the Limits of Choice’ in Sarah M Creighton and Lih-Mei Lao (eds), *Female Genital Cosmetic Surgery: Solution to What Problem?* (Cambridge University Press 2019) 78; See also Clare Chambers, ‘Are Breast Implants Better than Female Genital Mutilation? Autonomy, Gender Equality and Nussbaum’s Political Liberalism’ (2004) 7 Critical Review of International Social and Political Philosophy 1.

135 Proudman (n 7) 147.

136 Dustin and Phillips (n 21) 417.

137 ibid 417.

138 HL Deb 15 May 1985, vol 463, col 1237.

139 Explanatory Notes to Female Genital Mutilation Act 2003, para 6.

140 Dustin and Phillips (n 21) 417.

141 House of Commons Home Affairs Committee, ‘Female Genital Mutilation: The Case for a National Action Plan. Second Report of Session 2014-2015’, 14.

142 House of Commons Home Affairs Committee, ‘Female Genital Mutilation: Abuse Unchecked’ (2016) 17.

143 Nuffield Council on Bioethics, ‘Cosmetic Procedures: Ethical Issues’ (2017) xx; British Medical Association (n 94) 4; Royal College of Obstetricians and Gynaecologists, ‘Ethical Opinion Paper: Ethical Considerations in Relation to Female Genital Cosmetic Surgery (FGCS)’ (2013) 2; Royal College of Surgeons, ‘Cosmetic Surgery Standards FAQ’ <https://www.rcseng.ac.uk/standards-and-research/standards-and-guidance/service-standards/cosmetic-surgery/faq/> accessed 23 February 2021.

144 Clare Dyer, ‘London GP Is Cleared of Practising Female Genital Mutilation’ (2014) 348 BMJ 1.

145 ibid.

146 ibid.

147 Royal College of Obstetricians and Gynaecologists (n 143) 8.

148 David Veale and Joe Daniels, ‘Cosmetic Clitoridectomy in a 33-Year-Old Woman’ (2012) 41 Archives of Sexual Behavior 725.

149 ibid 726.

150 ibid 729.

151 ibid.

152 Proudman (n 7) 147.

153 Clare Dyer, ‘Doctor Alleged to Have Performed “Designer Vagina” Surgery Won’t Be Prosecuted’ [2016] BMJ i6390.

154 Martin Bentham, ‘Doctor Cleared over FGM Says Women Should Be Free to Have Intimate Surgery’ *Evening Standard* (London, 28 February 2017).

155 ibid.

156 Royal College of Obstetricians and Gynaecologists, ‘Female Genital Mutilation and Its Management’ (2015) 10–20.

157 Royal College of Obstetricians and Gynaecologists (n 143) 8.

158 Crown Prosecution Service, ‘Female Genital Mutilation Prosecution Guidance’.

159 ibid.

160 ibid.

161 Laville (n 22).

162 Proudman (n 7) 68.

163 Sandra Laville, ‘First FGM Prosecution: How the Case Came to Court’ *The Guardian* (4 February 2015).

164 Proudman (n 7) 68.

165 Haroon Siddique, ‘FGM Charges against Doctor Criticised by Obstetricians and Gynaecologists’ *The Guardian* (27 March 2014).

166 Crown Prosecution Service, ‘Written Evidence Submitted by Crown Prosecution Service’ (2014) para 9 <https://committees.parliament.uk/writtenevidence/48884/html/> accessed 13 November 2025.

167 ibid para 14.

168 ibid para 15.

169 ibid para 28.

170 ibid para 29.

171 ibid.

172 Cabinet Office, ‘Queen’s Speech 2014: What It Means for You’ (4 June 2014) para 10 <https://www.gov.uk/government/publications/queens-speech-2014-what-it-means-for-you/queens-speech-2014-what-it-means-for-you> accessed 13 November 2025.

173 Home Affairs Committee, ‘Female Genital Mutilation: The Case for a National Action Plan. Second Report of Session’ HC (2014-2015) Ch 4, para 97.

174 Girl Summit, ‘The Girl Summit Charter on Ending FGM and Child, Early and Forced Marriage’ (2014).

175 Serious Crime Bill Deb 20 January 2015, col 124.

176 HL Deb 5 January 2015, vol 590, col 75; HL Deb 16 June 2014, vol 754, col 647.

177 Serious Crime Bill 20 January 2015, col 126.

178 ibid col 124.

179 ibid co 121.

180 ibid col 127, col 128.

181 ibid col 131.

182 Home Office, ‘Introducing Mandatory Reporting for Female Genital Mutilation - A Consultation’ (2014).

183 Crown Prosecution Service, Written Evidence Submitted by Crown Prosecution Service (n 166) para 29.

184 Home Office (n 182) para 1.14.

185 Letter from Karen Bradley to Seema Malhotra MP (12 February 2015); the amendments also included the introduction of a clause which conferred powers to the Secretary of State to issue guidance on FGM. See HM Government, ‘Multi-Agency Statutory Guidance on Female Genital Mutilation’ (2020).

186 Home Office, ‘Mandatory Reporting of Female Genital Mutilation—Procedural Information’ (2015).

187 British Association of Social Workers, ‘FGM: Social Workers Should Not Take on the Role of the Police’ <https://basw.co.uk/articles/fgm-social-workers-should-not-take-role-police> accessed 7 August 2025.

188 House of Commons Affairs Committee, ‘Oral Evidence: Female Genital Mutilation, HC 1091’ (2014) 1.

189 British Medical Association, ‘Written Evidence Submitted by the British Medical Association (SC 08)’ (2015) para 8; See also M McCartney, ‘Disrespecting Confidentiality Isn’t the Answer to FGM’ (2015) 351 BMJ 1.

190 British Medical Association (n 189) para 5.

191 Home Office (n 182) para 1.15.

192 British Medical Association (n 189) para 5.

193 General Medical Council, ‘Protecting Children and Young People’ (2012).

194 Home Office (n 182) para 1.15.

195 HL Deb 5 January 2015, vol 590, col 75.

196 Royal College of Nursing, ‘Written Evidence Submitted by the Royal College of Nursing (RCN) (SC 04)’ (2015) para 6.1.

197 HC Deb Friday 21 March 2003, vol 40, col 1198.

198 Royal College of General Practitioners, ‘Introduction of Requirement for GPs to Submit Data under the FGM Enhanced Dataset Information Standard’; Department of Health, ‘FGM Prevention Programme: Understanding the FGM Enhanced Dataset: Updated Guidance and Clarification to Support Implementation’ (2015) 3.

199 Royal College of Obstetricians and Gynaecologists (n 156) Annex 1, 21.

200 NHS Digital, ‘Female Genital Mutilation: Official Statistics in Development’ <https://digital.nhs.uk/data-and-information/publications/statistical/female-genital-mutilation> accessed 13 November 2025.

201 Susan Bewley and others, ‘Mandatory Submission of Patient Identifiable Information to Third Parties: FGM Now, What Next?’ (2015) 351 BMJ 1.

202 ibid 1; See also Karen McVeigh, ‘FGM: Reporting of Cases among Children Becomes Mandatory’ *The Guardian* (20 October 2015).

203 NHS England Digital, ‘Female Genital Mutilation (FGM) Enhanced Dataset: Consultation 2024’ <https://digital.nhs.uk/data-and-information/clinical-audits-and-registries/female-genital-mutilation-datasets/female-genital-mutilation-fgm-enhanced-dataset-overview-of-2024-consultation/fgm-consultation-2024> accessed 7 August 2025.

204 Martina Larsson and others, ‘An Exploration of Attitudes towards Female Genital Mutilation (FGM) in Men and Women Accessing FGM Clinical Services in London: A Pilot Study’ (2018) 38 Journal of Obstetrics and Gynaecology 1005.

205 Erika Rackley and Rosemary Auchmuty, ‘The Case for Feminist Legal History’ (2020) 40 Oxford Journal of Legal Studies 878, 879.

206 Amy Abdelshahid, Kate Smith and Khadra Habane, ‘Do No Harm’. Lived Experiences and Impacts of FGM Safeguarding Policies and Procedures. Bristol Study’ (2021).

